# Supplementary material for: Prospective assessment of malaria infection in a semi-isolated Amazonian indigenous Yanomami community: Transmission heterogeneity and predominance of submicroscopic infection
Source: PLoS One. 2020 Mar 19;15(3):e0230643. doi: 10.1371/journal.pone.0230643 (PMC7081991; doi:10.1371/journal.pone.0230643)
Supplement: S1 File — (DOCX) [file pone.0230643.s001.docx]

**Prospective assessment of malaria infection in a semi-isolated Amazonian indigenous Yanomami community: transmission heterogeneity and predominance of submicroscopic infection**

Daniela Rocha Robortella^1,2^, Anderson Augusto Calvet^3^, Lara Cotta Amaral^2^, Raianna Farhat Fantin^2^, Luiz Felipe Ferreira Guimarães^2^, Michelle Hallais França Dias^2^, Cristiana Ferreira Alves de Brito^2^, Tais Nobrega de Sousa^2^, Mariza Maia Herzog^3^, Joseli Oliveira-Ferreira^3^*, Luzia Helena Carvalho^1,2^*.

**1** Universidade Federal de Minas Gerais (UFMG), Departamento de Parasitologia, Belo Horizonte, Brazil, **2** Instituto René Rachou (FIOCRUZ-MINAS), Belo Horizonte, Brazil, **3** Instituto Oswaldo Cruz, Fundação Oswaldo Cruz (IOC/FIOCRUZ), Rio De Janeiro, Brazil.

**Molecular protocols**

**Real-time PCR for amplification of the ABO blood group**

The gene coding for ABO blood group was amplified by real-time PCR using the following primer pair in the reaction, 516S and 926AS (**Table 1**). All PCR reactions were carried out in a total reaction volume of 10 µl (5 μL of SYBR® Green PCR Master Mix (Applied Biosystems), 0.5 μM of each primer (IDT) and 1 µL of DNA), with the following cycling conditions: 95˚C for 5 min, then 40 cycles of 30 s of denaturation at 95˚C, 30 s of annealing at 68˚C and 1 min extension at 72˚C. Amplification was followed by a melting program consisting of 15 s at 95°C, 20 s at 60°C and 95°C over the final 15 seconds. All real-time PCR assays was standardized and performed on the 7500 Real-Time PCR System (Applied Biosystems, software version 2.0.5) of the IRR Real-Time PCR Platform.

**Table 1.** Primers (516S and 926AS) described by Olsson *et al.* (1998).

| **Target** | **Primers names** | **5'-3'** | **T_m_°C*** |
| --- | --- | --- | --- |
| ABO | 516S (*foward*)  926AS (*reverse*) | GCTGGAGGTGCGCGCTAC  TACTTGTTCAGGTGGCTCTCGTC | 87.5 - 88.5°C |

*Melting temperature

**Amplification of non-ribosomal targets (Pvr47/Pfr364)**

**Real-time PCR (NR-qPCR)**

In order to amplified non-ribosomal targets for the detection of *P. vivax* (Pvr47) and *P. falciparum* (Pfr364), this assay was performed in 10 μL volumes containing 2 μL of DNA and 5 μL of TaqMan Universal PCR Master Mix (Applied Byosystems). For Pvr47 amplification was used 50 nM of forward primer, 900 nM of reverse primer and 250 nM of probe (**Table 2**). For Pfr364, 900 nM of forward primer, 300 nM of reverse primer and 150 nM of probe were used (**Table 2**), as previously described for Amaral *et al*. (2019). The PCR assays were performed using the automatic thermocycler ViiA7 Real-Time PCR System (Thermo Fisher Scientific) and the following cycling parameter: a pre-incubation and initial denaturation, respectively, at 50 °C for 2 min and 95 °C for 10 min, followed by 40 cycles of denaturation at 95 °C for 15 s, primers annealing at 52 °C for 1 min, and extension at 60 °C for 1 min. The fluorescence acquisition was performed at the end of each extension step. The cycle threshold (Ct) values of 37 and 38 (Ct≤37 or Ct≤38) were used to define positivity to *P. vivax* and *P. falciparum*, respectively.

**Table 2**. Primers Pvr47 and Pfr364, described by Amaral *et al.* (2019).

| **Target** | **Primers/probes names** | | **5’-3’** |
| --- | --- | --- | --- |
| *P. vivax* | Pvr47 (*foward*) | TCCGCAGCTCACAAATGTTC | |
|  | Pvr47 (*reverse*) | ACATGGGGATTCTAAGCCAATTTA | |
|  | Pvr47 (*probe*) | **HEX**TCCGCGAGGGCTGCAA | |
| *P. falciparum* | Pfr364 (*foward*) | ACTCGCAATAACGCTGCAT | |
|  | Pfr364 (*reverse*) | TTCCCTGCCCAAAAACGG | |
|  | Pfr364 (*probe*) | **FAM**GGTGCCGGG GGTTTCTACGC | |

**Amplification of ribosomal targets (18SrRNA)**

**Real-time PCR (R-qPCR)**

In this assay, conserved sense and antisense primers, associated with the species-specific probes, distinguish the *Plasmodium* species (**Table 3**), being this protocol realized in three mixes separated according to the species. The protocol was standardized with a final volume of 12.5 μL (2 μL DNA and 6,25 μL of TaqMan Universal PCR Master Mix (Applied Byosystems), with the concentrations of the primers and probes established by Rougemont et al., (2004), being 200 nM for each primer and 80 nM for the respective probes). The PCR conditions consisted of: 50°C for 2 min, 95°C for 10 min, 95°C for 15 s and 60°C for 1 minute, resulting in a final constant of 45 cycles. The sample was considered positive by identifying the Ct at which normalized reporter dye emission raised above background noise. If the fluorescent signal did not increase within 40 cycles (Ct 40), the sample was considered negative.

**Table 3**. Primers (Plasmo 1 and 2) and probes (Viv, Fal, Mal) described by Rougemont *et al.* (2004).

| **Target** | **Primers/probes names** | | **5’-3’** |
| --- | --- | --- | --- |
| *Plasmodium sp.* | Plasmo1 (*foward*)  Plasmo2 (*reverse*) | | GTTAAGGGAGTGAAGACGATCAGA  AACCCAAAGACTTTGATTTCTCATAA |
| *P. vivax* | Viv (*probe*) | **VIC**AGCAATCTAAGAATAAACTCCGAAGAGAAAATTCT**MGBNFQ** | |
| *P. falciparum* | Fal (*probe*) | **FAM**AGCAATCTAAAAGTCACCTCGAAAGATGACT**MGBNFQ** | |
| *P. malariae* | Mal (*probe*) | **FAM**CTATCTAAAAGAAACACTCAT**MGBNFQ** | |

**Nested-PCR**

Samples were amplified using a Nested-PCR protocol adapted from the original protocol described by Snounou et al. (1993). The same primers described in the original study were used in this study (**Table 4**). Briefly, all PCR reactions were performed in 20 μL volumes containing 0.25 μM of each primer (IDT), 1 0μL of Master Mix (Promega) and 0.8 μL of DNA (for the second reaction, 0.8μL of the primary product was used as template). The PCR assays were performed using an automatic thermocycler (PTC-100TM v.7.0) (MJ Research Inc, USA) and the following cycling parameters were used: an initial denaturation at 95°C for 5 min, 58°C for 2 min and 72°C for 2 min followed by 24 cycles of denaturation at 94°C for 1 min, annealing at 58°C for 2 min and extension at 72°C for 2 min, followed by a final annealing incubation at 58°C for 2 min and extension at 72°C for 5 min. The cycling parameters for the second round of PCR were the same as the first reaction, but instead 29 cycles of amplification were used. The amplified products were detected by ethidium bromide staining following agarose 2% gel electrophoresis (Invitrogen).

**Table 4**. Primers (rPLU5/6, rVIV, rFAL and rMAL) described by Snounou *et al.* (1993).

| **Target** | **Primers names** | **5’-3’** | **Amplicons** |
| --- | --- | --- | --- |
| *Plasmodium sp.* | rPLU5 (*foward*) | CCTGTTGTTGCCTTAAACTTC | 1,2Kb |
|  | rPLU6 (*reverse*) | TTAAAATTGTTGCAGTTAAAACG |  |
| *P. vivax* | rVIV1 (*foward*) | CGCTTCTAGCTTAATCCACATAACTGATAC | 120pb |
|  | rVIV2 (*reverse*) | ACTTCCAAGCCGAAGCAAAGAAAGTCCTTA |  |
| *P. falciparum* | rFAL1 (*foward*) | TTAAACTGGTTTGGGAAAACCAAATATATT | 205pb |
|  | rFAL2 (*reverse*) | ACACAATGAACTCAATCATGACTACCCGTC |  |
| *P. malariae* | rMAL1 (*foward*) | ATAACATAGTTGTACGTTAAGAATAACCGC | 144pb |
|  | rMAL2 (*reverse*) | AAAATTCCCATGCATAAAAAATTATACAAA |  |
