# Supplementary figures and images for: Prospective assessment of malaria infection in a semi-isolated Amazonian indigenous Yanomami community: Transmission heterogeneity and predominance of submicroscopic infection
Source: PLoS One. 2020 Mar 19;15(3):e0230643. doi: 10.1371/journal.pone.0230643 (PMC7081991; doi:10.1371/journal.pone.0230643)

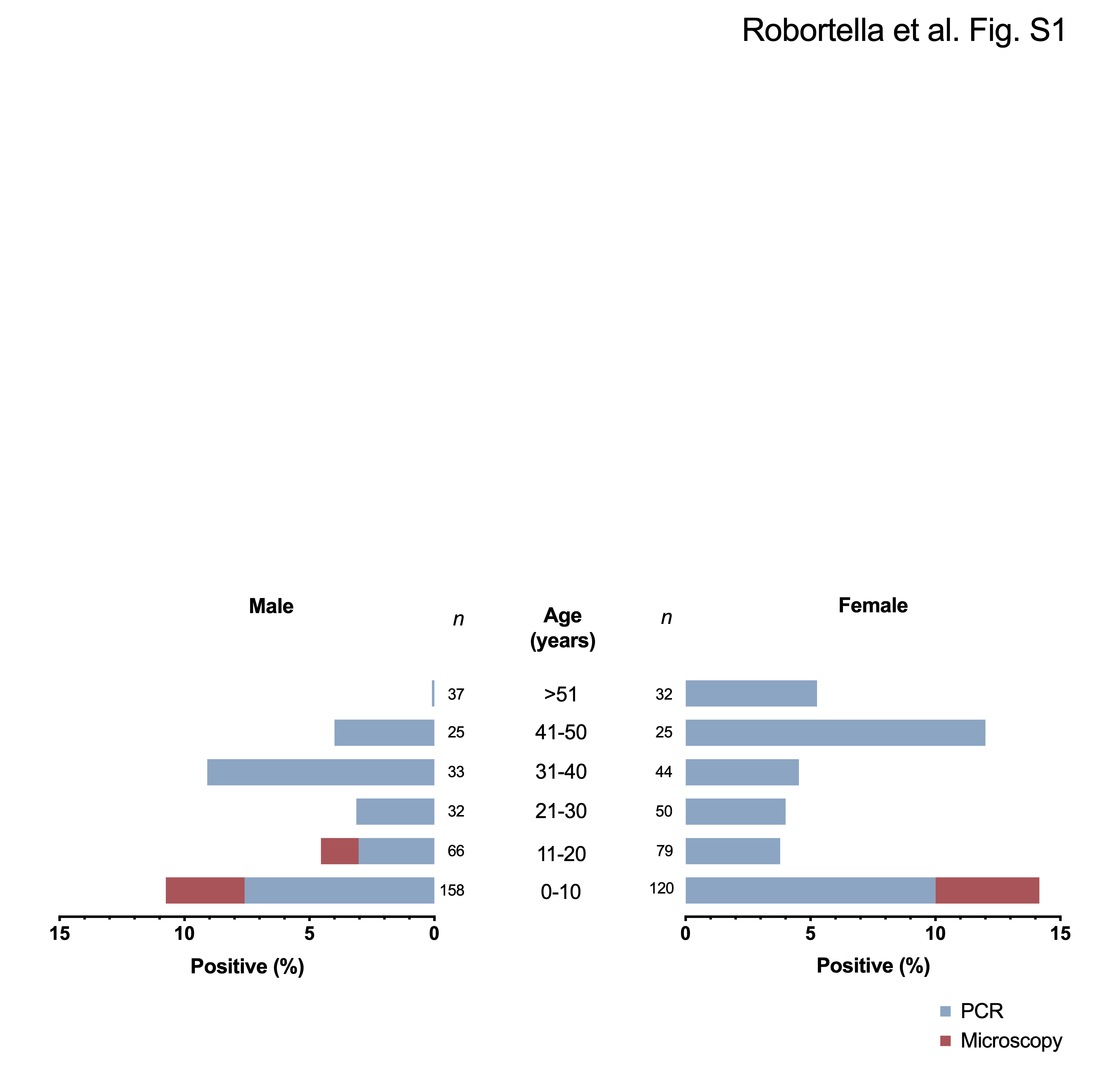

Supplement: S1 Fig — Malaria positivity was defined by PCR-based protocols (PCR) or by conventional light microscopy (Microscopy). The number (n) of individuals in each age group is represented in the respective bars. (TIFF) [file pone.0230643.s002.tiff]

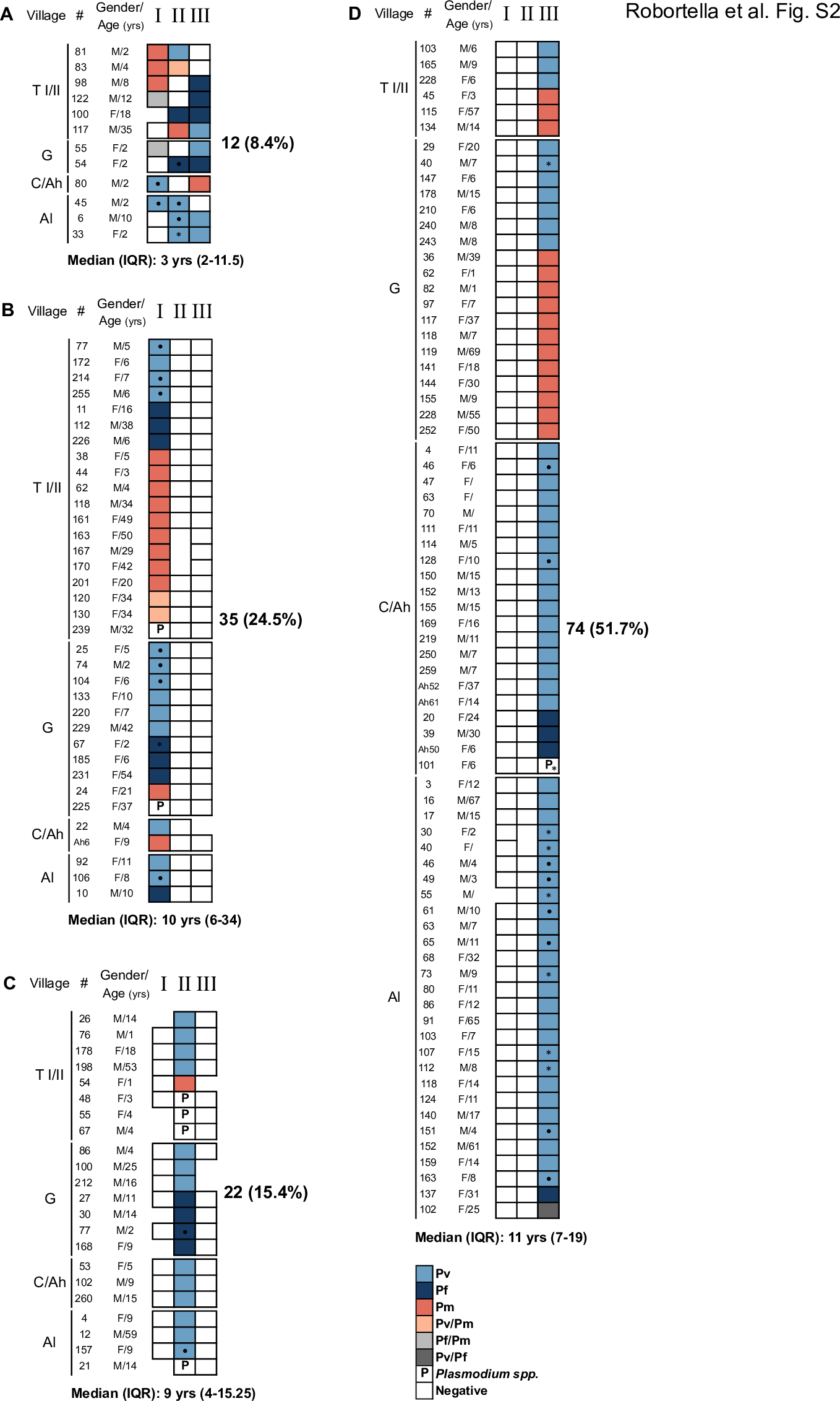

Supplement: S2 Fig — Each row represents a single individual coded (#) and grouped according to their profile of positive samples in each of the three cross-sectional surveys (columns I, II and III, respectively, of the colored matrix). (A) shows individuals who were positive in at least two of the three cross-sectional surveys (n = 12). (B), (C) and (D) show individuals who were positive in only one of the three cross-sectional surveys: either (B) the 1st baseline survey (I; n = 35), (C) the 2nd survey (II, n = 22) or (D) the 3rd survey (III; n = 74). Species-specific PCR positivity is represented using different colors, as indicated in the legend: P. vivax (Pv) in light blue; P. falciparum (Pf) in dark blue; P. malariae (Pm) in orange; and mixed infections by the other different colors. The dark dot (•) inside each square indicates positivity by microscopy as well as PCR, while the asterisks (*) indicate positivity only by microscopy. Individual age, gender and place of dwelling were included in the left part of figure. Each village was coded as according to the legend of Fig 5. (TIF) [file pone.0230643.s003.tif]

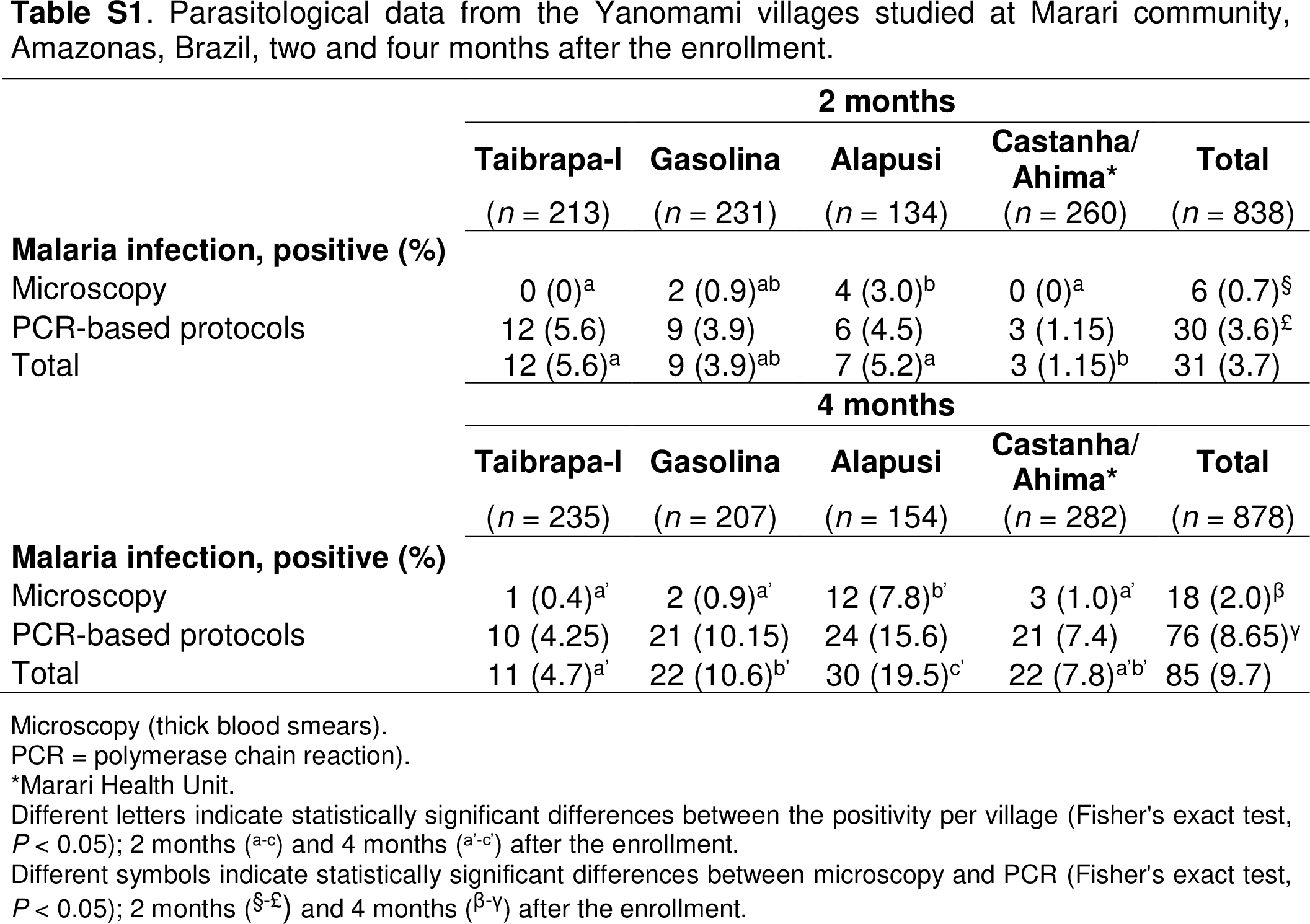

Supplement: S1 Table — (TIF) [file pone.0230643.s004.tif]
